# Effects of first exposure to plain cigarette packaging on smoking behaviour and attitudes: a randomised controlled study

**DOI:** 10.1186/s12889-015-1586-8

**Published:** 2015-03-13

**Authors:** Olivia M Maynard, Ute Leonards, Angela S Attwood, Linda Bauld, Lee Hogarth, Marcus R Munafò

**Affiliations:** MRC Integrative Epidemiology Unit (IEU) at the University of Bristol, Bristol, UK; UK Centre for Tobacco and Alcohol Studies, Bristol, UK; School of Experimental Psychology, University of Bristol, 12a Priory Road, Bristol, BS8 1TU United Kingdom; Institute of Social Marketing, University of Stirling, Stirling, UK; Department of Psychology, University of Exeter, Exeter, UK

**Keywords:** Plain cigarette packaging, Standardised packaging, Smoking, Randomised controlled trial

## Abstract

**Background:**

Plain packaging requires tobacco products to be sold in packs with a standard shape, method of opening and colour, leaving the brand name in a standard font and location. We ran a randomised controlled trial to investigate the impact of plain packaging on smoking behaviour and attitudes.

**Methods:**

In a parallel group randomised trial design, 128 daily smokers smoked cigarettes from their usual UK brand, or a plain Australian brand that was closely matched to their usual UK brand for 24 hours. Primary outcomes were number of cigarettes smoked and volume of smoke inhaled per cigarette. Secondary outcomes were self-reported ratings of motivation to quit, cigarette taste, experience of using the pack, experience of smoking, attributes of the pack, perceptions of the health warning, changes in smoking behaviour, and views on plain packaging.

**Results:**

There was no evidence that pack type had an effect on either of the primary measures (*p*s > 0.279). However, smokers using plain cigarette packs rated the experience of using the pack more negatively (−0.52, 95% CI −0.82 to −0.22, *p* = 0.001), rated the pack attributes more negatively (−1.59, 95% CI −1.80 to −1.39, *p* < 0.001), and rated the health warning as more impactful (+0.51, 95% CI 0.24 to 0.78, *p* < 0.001).

**Conclusions:**

Plain cigarette packs reduce ratings of the experience of using the cigarette pack, and ratings of the pack attributes, and increase the self-perceived impact of the health warning, but do not change smoking behaviour, at least in the short term.

**Trial registration:**

Current Controlled Trials ISRCTN52982308. Registered 27 June 2013.

**Electronic supplementary material:**

The online version of this article (doi:10.1186/s12889-015-1586-8) contains supplementary material, which is available to authorized users.

## Background

Mandatory plain (“standardised”) packaging requires cigarettes and hand-rolling tobacco to be sold in packs with a standard pack shape, method of opening and colour, leaving only the brand name in a standard font and location. In December 2012, Australia became the first country in the world to introduce plain cigarette packaging. The United Kingdom (UK) government are currently discussing the issue.

Evidence to date, predominantly comprising survey and observational studies, suggests that plain cigarette packaging increases attention to cigarette pack health warnings [1,2], prevents the use of misleading pack characteristics, such as pack colouration to donote cigarette strength [3], and makes the pack of cigarettes less appealing, both in terms of the pack itself [4-6] and the taste and quality of the cigarettes inside [7-9]. Observational studies requiring smokers to use branded and plain cigarette packs for a period of weeks have found that plain cigarette packaging is associated with increased negative feelings about the cigarette pack and about smoking, increased avoidant behaviours towards the pack (such as keeping it out of sight) and increased prevalence of cessation behaviours (such as smoking less around others and forgoing cigarettes) [5,6]. In post-study interviews, the first of these studies [5] found that these effects of plain cigarette packaging, such as avoidant behaviours and reduced cigarette consumption, occurred most frequently among female smokers and as a result, the second study recruited only female smokers [6]. A cross-sectional observational study from Australia during the period of implementation of plain cigarette packaging found that smokers using plain cigarette packs perceived their cigarettes to be of lower quality and less satisfying, had thought about quitting more often, and rated quitting as a higher priority in their lives than those still using branded cigarette packs [10]. Furthermore, anecdotal evidence which emerged from Australia soon after the introduction of plain cigarette packaging suggested that cigarettes from plain cigarette packs tasted worse than those from branded cigarette packs [11]. Finally, an analysis of calls to an Australian smoking cessation helpline found an increase in calls after the introduction of plain cigarette packaging [12].

While this research provides important information on the impact of plain cigarette packaging on attitudes to smoking and the cigarette pack, no research has directly measured the impact of plain cigarette packaging on smoking behaviour, used genuine plain cigarette packs, or used an experimental design. In the present study, we overcame these previous shortcomings and assessed whether plain, as compared with branded cigarette packaging, resulted in changes in smoking behaviour, as well as attitudes towards smoking, when using these packs over 24 hours, in a randomised controlled trial using Australian plain cigarette packs.

## Methods

### Study design and participants

The study was conducted at the University of Bristol. First randomisation was on March 3, 2013, and last follow-up on December 9, 2013. The published protocol [13] describes the procedures in detail and no changes to the trial design or method were made after trial commencement. In brief, 128 regular daily smokers, defined as: 1) smoking every day of the week, 2) smoking between 5 and 20 cigarettes a day, 3) smoking within one hour of waking, and 4) not planning to quit smoking within the next six months, were recruited. Participants were also required to be aged between 18 and 40 years, to predominantly smoke one of the specific brands of cigarettes available in the study (Marlboro Gold, Marlboro Red, Dunhill Red, Benson and Hedges Gold, Benson and Hedges Silver), to be in good physical and mental health, to not be taking any psychiatric medication or illicit drugs, and (if female) to not be pregnant. Each of these were assessed with a pre-study self-report online screening. Participants were recruited from the staff and students at the University of Bristol and the general population, through existing email lists, poster and flyer advertisements, online and by word of mouth. Prior to arranging a testing session, potential participants completed an online screening questionnaire to assess eligibility for the study.

Participants first attended a baseline day, where they completed questionnaire measures and provided blind taste ratings of a cigarette from a branded and a plain cigarette pack. Participants were then randomly allocated to either a branded or plain cigarette pack and were given instructions for the following *ad libitum* smoking day. The smoking day started the morning after the baseline day and participants smoked cigarettes from the cigarette pack provided to them and through a smoking topography monitor for the entire day. The day after the smoking day, participants attended a final test day, during which they returned any remaining cigarettes and the smoking topography monitor and completed questionnaires about the smoking day and further taste ratings.

Ethics approval was granted by the Faculty of Science Research Ethics Committee at the University of Bristol (Ethics Approval Code 310113607). The study was conducted according to the revised Declaration of Helsinki 2013 and Good Clinical Practice guidelines. All participants provided fully informed written consent prior to testing.

### Randomisation

Participants were randomised to receive either a plain or branded cigarette pack of their usual brand of cigarettes to smoke for the smoking day. Smokers randomised to the branded and plain cigarette pack conditions were matched by gender, with equal numbers of males and females in each condition. The lead researcher was blind to the condition assigned to participants until the participant returned on the final test day. To perform the randomisation, the lead researcher, who enrolled participants, contacted an experimental collaborator with the participant’s preferred brand of cigarettes and the participant’s gender. The collaborator then used random number generator software, along with a pre-assigned code, to allocate the participant to the branded or plain cigarette pack condition. A pack of the assigned cigarettes was then placed into a concealed envelope labelled with the participant’s anonymised identification number.

### Materials

#### Cigarette packs

**Figure 1:**
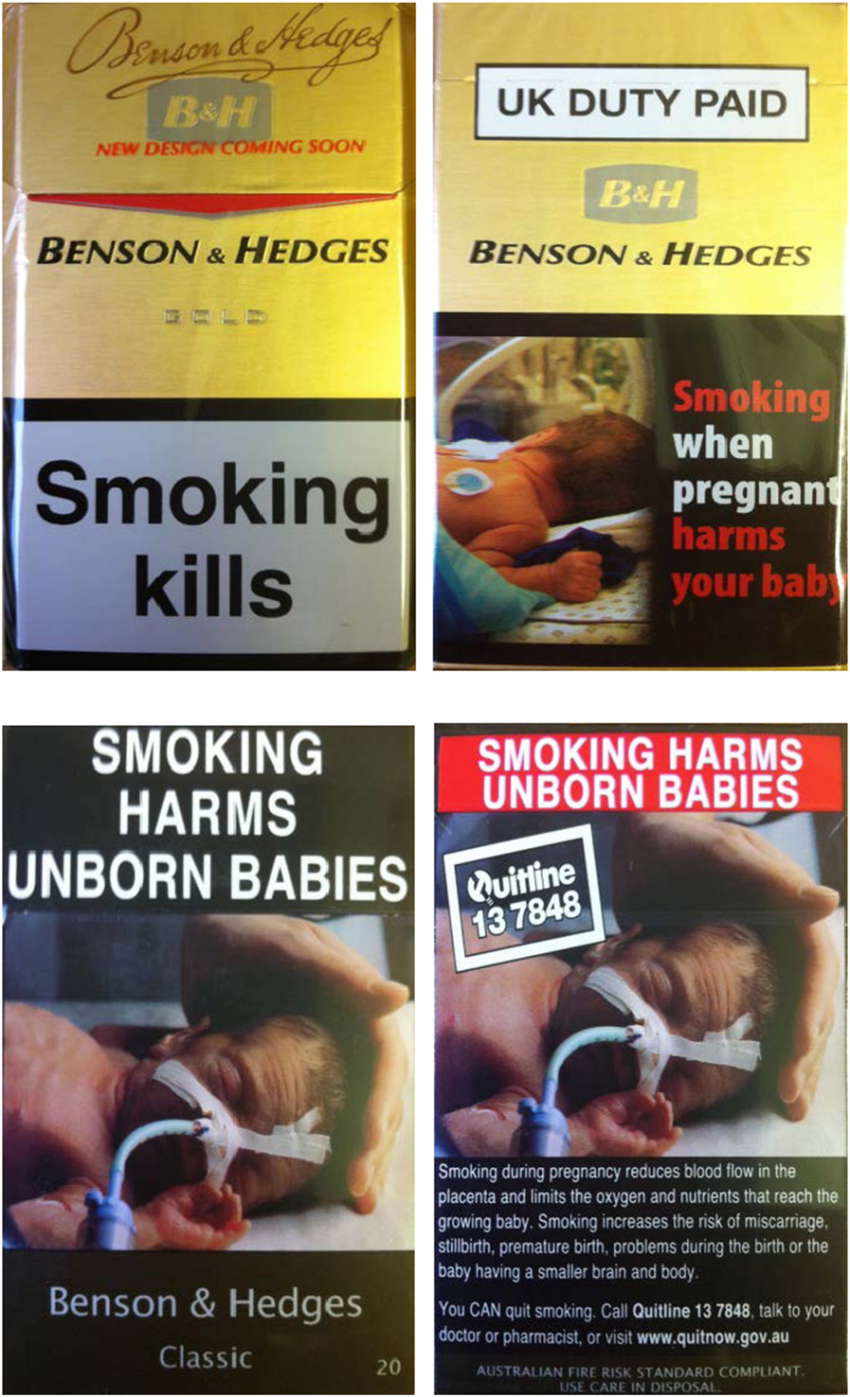
**Examples of the front and back of UK branded and Australian plain packs.**

Participants were given either their usual UK branded cigarette pack of cigarettes or an Australian plain cigarette pack of cigarettes which matched their preferred UK brand, to smoke for the smoking day. Since they originated from two different countries, packs differed in the shape, size and format of the health warning; however, they were selected so that both presented a message related to the effects of prenatal tobacco exposure on offspring. Examples of the Australian plain and UK branded cigarette pack cigarettes used are shown in Figure 1. This health warning was selected as it was this warning which was most similar between the two countries.

#### Topography monitor

A battery-operated, hand-held CreSS Pocket topography monitor (CReSS; Borgwaldt KC, Hamburg, Germany) was used to record participants’ smoking behaviour. For each cigarette smoked, the monitor recorded the date, time, start and end of smoking, puffs per cigarette, puff volume and puff duration.

#### Questionnaires

To assess the taste of the cigarettes smoked at the blind ‘tasting’ on the baseline day, participants were asked “How did this cigarette taste”, and when they returned on the final test day, participants were asked “How did the cigarettes in the pack given to you yesterday taste”. To answer these questions, participants were required to report their agreement with the statements “The taste of this cigarette was strong/harsh/dry/stale/dull/dirty”, each on a seven-point scale between “Strongly disagree” to “Strongly agree”. Participants also reported on a seven-point scale between “Much better” to “Much worse” the answer to the question “Compared to my usual cigarette, the taste of this cigarette is…”.

At the final test day, participants also answered a series of questions regarding their experiences on the smoking test day. To assess ‘*Experience of smoking*’, participants were asked, “To what extent did you experience the following as you smoked the cigarettes? a) Enjoyment, b) Satisfaction, c) Acceptance”. To assess *‘Experience of using the pack’,* participants were asked “To what extent did you experience the following about the cigarette pack? a) Embarrassment, b) Shame, c) Acceptance”. To assess ‘*Rating of cigarette pack attributes’*, that is, participants’ perceptions of the packs, participants were asked to “Rate the cigarette pack on the following attributes: a) Style, b) Fashion, c) Cheapness, d) Coolness, e) Attractiveness, f) Quality, g) Appeal”. To assess participants’ ‘*Rating of the health warning’*, participants were asked to “Rate the health warning on the following attributes: a) Noticing, b) Seriousness, c) Believability, d) Awareness of health risks”. To assess *‘Experience of using topography monitor’,* participants were asked “To what extent did you experience the following about the monitor you were asked to smoke your cigarettes through? a) Embarrassment, b) Shame, c) Acceptance”. Participants responded to each of these questions on a five-point scale, with higher scores indicating higher agreement with each of the statements. Mean responses across the sub-questions were then calculated in order to calculate an overall response for each of the five questions. ‘*Changes in behaviour’*, was also assessed by asking participants “During the 24 hours when you smoked cigarettes from the pack given to you in the experiment, did you 1) Stub out a cigarette early, 2) Forgo a cigarette (i.e. not have a cigarette when you normally would have, 3) Keep the pack out of sight, 4) Cover the pack, 5) Smoke less around others, 6) Think about cutting down, 7) Think about quitting in the next few weeks, 8) Thinking about quitting within a year”. Each of these statements were answered with binary (yes/no) responses. ‘Yes’ responses were summed to create an overall score of smoking behaviour. With the exception of the questions relating to the topography monitor, each of these questions were taken from previous studies assessing smoking behaviour when using cigarettes in plain cigarette packs [5,6]. Finally, participants were also asked to report their ‘*Attitudes to plain packs’* by answering the following three questions “Do you think plain packaging would make you smoke fewer cigarettes?”, “Do you think plain packaging would help you to quit smoking?” and “Do you think plain packaging would prevent children from starting smoking?” Each of these questions were answered on a four-point scale, with higher scores indicating higher agreement with each of the questions.

### Procedure

#### Baseline day

On arrival at the baseline day, participants re-read the information sheet and provided informed consent. Participants were then asked to report the number of cigarettes they had smoked the previous day, and the number of minutes since their last cigarette. Smoking status was verified using a breath Carbon Monoxide (CO) test. Participants completed baseline questionnaires assessing contemplation of quitting smoking (Contemplation Ladder) [14], smoking urges (Questionnaire of Smoking Urges; QSU-brief) [15] and nicotine dependence (Fagerström Test for Nicotine Dependence; FTND) [16]. Participants next completed the cigarette ‘taste test’, where they were presented with two cigarettes to smoke, in counterbalanced order, under double-blind conditions, one taken from a plain cigarette pack of their preferred brand of cigarettes and the other from a branded cigarette pack. Given anecdotal evidence indicating poor taste of cigarettes from plain cigarette packs, taste ratings were obtained to provide a comparison with taste ratings at the final test day, when participants knew whether the cigarettes were from a plain or branded cigarette pack. In a purpose-built smoking laboratory, participants were asked to take three puffs from each of the two cigarettes in turn and record how they tasted (see ‘Questionnaires’ section). After completing the ratings, participants were given a glass of water to cleanse their palate and waited five minutes before completing the same procedure with the second cigarette.

After the taste test, participants were provided with a topography monitor and given verbal instruction on how to operate it. They were then instructed to practice using the monitor with a cigarette in the smoking laboratory, during which participants typically smoked either one or two cigarettes of their usual brand (data not analysed). Participants were then presented with a sealed envelope, which contained either a plain or branded cigarette pack of their usual brand, as described in the randomisation procedure. Participants were blind to the pack type given to them until they opened the envelope, which they were instructed to do the following morning. Participants were asked to use this pack of cigarettes and the monitor for the entirety of that day. Participants were informed that the main purpose of the study was to investigate factors which influence smoking behaviour (*i.e.,* not specifically cigarette packaging) and were not informed that the cigarette pack was an integral aspect of the experiment. Participants were asked to return the pack with any remaining cigarettes on the final test day (to verify the number smoked). To ensure that all unsmoked cigarettes were returned, participants were told they would be able to take these with them at the end of the study.

#### Final test day

Approximately 48 hours after the baseline day, participants returned the smoking topography monitor and any un-smoked cigarettes, and completed the quitting smoking Contemplation Ladder [14]. Response options on the Contemplation Ladder range from 0 to 10, where “0” corresponds with the response “I have no thoughts about quitting smoking” and “10” corresponds with the response “I am taking action to quit smoking”. To further verify the data obtained from the topography device, participants also self-reported the number of cigarettes they smoked during the smoking day, gave details of any cigarettes not smoked from the pack given to them or through the monitor, and any unusual events about the previous day, which may have changed their smoking behaviour. Participants then rated the taste of the cigarettes they had been provided with, using the same questions as the baseline day, and completed the questionnaires described previously. Participants then completed a computer task assessing the degree to which branded and plain packs elicited tobacco seeking, the results of which are presented elsewhere [17]. Finally, after participants in both conditions had been shown pictures of Australian plain packs, participants were asked to report their attitudes to these packs, so as to ascertain whether 24-hour exposure to plain packaging reduced smokers’ negative attitudes to this packaging. On completing the experiment, participants were fully debriefed and given the opportunity to ask questions. Participants were reimbursed £30 for completing the study.

### Outcome measures

Primary outcomes were number of cigarettes smoked and volume of smoke inhaled per cigarette, as measured by a smoking topography monitor (number of cigarettes smoked was confirmed with a self-report measure). Secondary outcomes were self-reported ratings of motivation to quit smoking (as measured by the Quitting Contemplation Ladder), the cigarette taste test, experience of smoking, experience of using the pack, rating of pack attributes, rating of the health warning, changes in behaviour, and attitudes to plain cigarette packs.

### Statistical analysis

The sample size for the study was calculated based on the primary outcome of average volume of smoke inhaled per cigarette. Given the inelasticity of smoking behaviour, with daily smokers relatively stable with regards to the number of cigarettes they smoke per day, we hypothesised that volume of smoke inhaled would be a more sensitive measure of tobacco exposure than number of cigarettes smoked, given that this is known to be relatively plastic [14]. A reduction in volume of smoke inhaled would be meaningful if continued over an extended period. Pilot data using these topography monitors indicated a mean inhaled volume per cigarette of 500 mL (Standard Deviation (SD) = 100). Therefore, in order to detect a reduction in inhaled volume of 50 mL per cigarette (*i.e.,* equivalent to one fewer cigarette per day for a 10 a day smoker) with 80% power at an alpha level of 5%, we recruited 128 participants.

Linear regression was used to evaluate the effect of cigarette packaging (branded or plain) on the primary and secondary outcome measures. These analyses were conducted with and without adjustment for age, gender, heaviness of smoking and, where appropriate, corresponding baseline measures. Whether these effects differed between males and females was investigated by including appropriate interaction terms in the models. IBM SPSS Statistics 19 was used to analyse the data.

## Results

### Characteristics of participants

**Figure 2:**
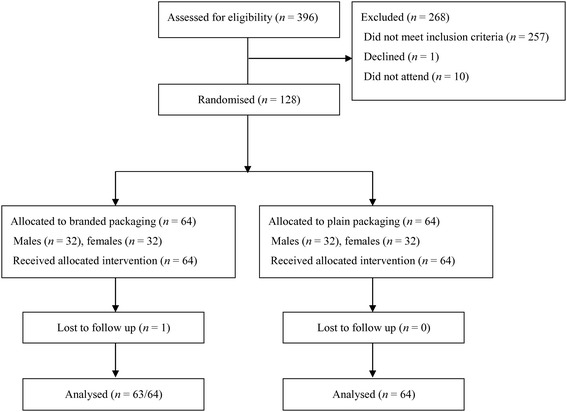
**CONSORT flow chart.**

Of 396 people who completed the initial assessment, 128 met the inclusion criteria and were recruited into the study, with 64 participants assigned to branded cigarette packs and 64 to plain cigarette packs (see Figure 2). Of the remaining 268 participants, 257 did not meet the inclusion criteria (the majority did not smoke one of the specific brands used in the study or failed to meet the smoking behaviour criteria [i.e. number of cigarettes smoked per day or time to first cigarette]), 10 failed to attend their allocated testing session and one participant declined to participate after completing the initial assessment.

One participant randomised to branded cigarette packaging did not provide secondary outcome data. Participants’ baseline characteristics were similar in both groups (see Table 1). There was no difference between smokers randomised to the branded cigarette pack versus plain cigarette pack condition with regard to their ratings of using the topography monitor (*t* = −1.16 _[125],_*p* = 0.246, branded: Mean (M) = 3.79, Standard Error (SE) = 0.11, plain: M = 3.98, SE = 0.08, with higher scores representing more acceptance of the monitor).

**Table 1:** **Baseline characteristics of participants**

	**Branded** ***n*** **= 64 Mean (SE)**	**Plain** ***n*** **= 64 Mean (SE)**
Age	21.09 (0.37)	21.66 (0.46)
Cigarettes smoked per day	10.05 (0.37)	10.14 (0.40)
Years daily smoker	4.42 (0.38)	5.19 (0.50)
Exhaled carbon monoxide*	9.85 (1.05)	9.11 (0.85)
Number of cigarettes smoked previous day	10.23 (0.45)	9.97 (0.46)
Minutes since last cigarette	44.25 (5.16)	59.05 (11.32)
Quit contemplation	4.22 (0.13)	4.28 (0.10)
Questionnaire of Smoking Urges - Brief	34.28 (1.50)	34.55 (1.31)
Fagerström Test of Nicotine Dependence	3.60 (0.22)	3.58 (0.19)

*Two participants in the branded cigarette pack condition did not provide a carbon monoxide sample.

### Primary outcomes

As shown in Table 2, smokers randomised to the plain cigarette pack smoked on average fewer cigarettes than those randomised to the branded cigarette pack (−0.58 cigarettes, 95% Confidence Interval (CI) = −1.63 to +0.48, *p* = 0.28, branded: M = 10.86, plain: M = 10.34), and inhaled more smoke per cigarette (+54.78 mL, 95% CI −112.50 to +222.07, *p* = 0.52, branded: M = 765.15, plain: M = 817.26). However, in both cases the confidence intervals were wide and included the null, so that we cannot conclude that plain cigarette packs had any effect on smoking behaviour after first exposure. Interaction tests did not indicate any evidence that smoking behaviour differed for males and females (*p*s > 0.90).

**Table 2:** **Summary of linear regression results for final test day**

			**Unadjusted**				**Adjusted**			
	**Branded (** ***n*** **= 63) Mean (SE)**	**Plain (** ***n*** **= 64) Mean (SE)**	**B**	**Lower**	**Upper**	***P***	**B**	**Lower**	**Upper**	***P***
Primary outcomes										
Number of Cigarettes Smoked	10.86 (0.45)	10.34 (0.48)	−0.51	−1.81	+0.78	0.435	−0.58	−1.63	+0.48	0.279
Volume (mL) of Smoke Inhaled	765.15 (62.41)	817.26 (55.24)	+52.10	−112.70	+216.91	0.533	+54.78	−112.50	+222.07	0.518
Secondary Outcomes										
Quitting Contemplation Ladder	4.35 (0.15)	4.28 (0.12)	−0.07	−0.44	+0.31	0.719	−0.09	−0.31	+0.13	0.432
Taste Test	3.22 (0.10)	3.51 (0.12)	+0.29	−0.02	+0.59	0.068	+0.20	−0.08	+0.48	0.154
Experience of smoking	3.77 (0.09)	3.57 (0.10)	−0.20	−0.47	+0.07	0.140	−0.18	−0.45	+0.09	0.183
Experience of using the pack	4.63 (0.07)	4.13 (0.13)	−0.50	−0.80	−0.21	0.001	−0.52	−0.82	−0.22	0.001
Rating of pack attributes	3.52 (0.08)	1.91 (0.07)	−1.61	−1.82	−1.40	< 0.001	−1.59	−1.80	−1.39	< 0.001
Rating of the health warning	3.92 (0.11)	4.41 (0.08)	+0.49	+0.23	+0.76	< 0.001	+0.51	+0.24	+0.78	< 0.001
Change in behaviour	2.06 (0.21)	2.20 (0.20)	+0.14	−0.43	+0.71	0.629	+0.11	−0.45	+0.68	0.695
Attitudes to plain packs	6.66 (0.30)	6.21 (0.05)	−0.45	−1.27	+0.38	0.285	−0.39	−1.22	+0.44	0.350

### Secondary outcomes

Smokers randomised to the plain cigarette pack condition, as compared with those randomised to the branded cigarette pack condition, reported more negative experiences of using the pack (−0.52, 95% CI −0.82 to −0.22, *p* = 0.001), more negative ratings of the pack attributes (−1.59, 95% CI −1.80 to −1.39, *p* < 0.001), and that the health warning was more impactful (+0.51, 95% CI +0.24 to +0.78, *p* < 0.001). There was no clear evidence that plain cigarette packaging influenced the taste of the cigarette, changed self-reported behaviour, increased motivation to quit, changed the experience of smoking, or changed attitudes to plain cigarette packs (*p*s > 0 15). However, the point estimates for these outcomes were all in the direction of a beneficial effect (*e.g.,* more unpleasant taste). Again, interaction tests did not indicate any evidence that these results differed for males and females (*p*s > 0.18). These results are shown in full in Table 2 and results for each of the individual items which constitute the overall measures are presented in the Additional file 1: Table S1.

## Discussion

This is the first randomised controlled trial evaluating the effects of plain cigarette packaging on smoking behaviour and attitudes to smoking. No differences were observed between those randomised to branded and plain cigarette packs on the primary outcome measures of number of cigarettes smoked and the volume of smoke inhaled, suggesting that plain packaging may not influence actual smoking behaviour after a single exposure. The number of cigarettes smoked during the trial for participants in both conditions was slightly higher than that reported at baseline, although this effect is likely a result of underreporting by participants in the self-report baseline measure, a well-documented behaviour among smokers [18].

We did, however observe that smoking cigarettes from a plain cigarette pack for a 24-hour period had clear effects on ratings of the experience of using the pack, the pack attributes, and the impact of the health warning. Previous research has shown that more negative perceptions of smoking [19,20] and greater awareness of the health warning [21,22] can act as triggers to reduce smoking behaviour such as forgoing cigarettes and making quit attempts, both of which predict eventual successful quitting. It is therefore possible that these differences in attitudes may take longer than 24 hours to affect behaviour. We found no clear evidence that plain cigarette packaging influenced the taste of the cigarette, changed self-reported behaviour, increased motivation to quit, changed the experience of smoking, or changed attitudes to plain cigarette packs. However, for all of these measures, the effects observed were in the direction of a beneficial effect of plain packaging. Again, it is possible that it may take longer than 24 hours for plain packaging to affect these attitudes and behaviours.

There are some important strengths of our research design. In particular, we used genuine plain cigarette packs, which were imported into the UK specifically for the purpose of this study, taking advantage of their recent introduction in Australia in December 2012. Previous studies investigating plain cigarette packaging have required smokers to transfer their cigarettes into plain cigarette packs created by the researchers [6]. Accordingly, these packs did not include several of the features typically found on tobacco industry cigarettes, such as plastic wrap around the packs and foil inside the packs, and they did not use genuine brand names. It is possible that some of the negative attitudes to these packs observed in these earlier studies may thus be a result of these features, rather than a direct result of the plain cigarette packaging. Using genuine plain cigarette packs in the present study avoided these possible issues and increased the external validity of our study. Moreover, we used an experimental design, randomising participants to use branded or plain packs and rather than simply asking participants to report their smoking behaviour, we examined the effect of plain cigarette packaging on actual smoking behaviour over 24 hours as measured by a topography monitor.

There are also some limitations to our research design. Whilst the Australian plain cigarette packs and the branded UK cigarette packs were matched for brand, there were differences between the packs and cigarettes inside, such as the size and format of the health warnings, the constituent information on the pack, the design of the cigarettes themselves and the specific tobacco in the cigarettes. However, as plain cigarette packaging legislation will most likely be introduced alongside larger health warnings, similar to those on the Australian plain cigarette packs, using these plain cigarette packs likely increases the overall ecological validity of the study, although we are not able to determine how smokers would respond to genuine UK plain cigarette packs. Data on whether participants in the plain pack condition were aware they were using Australian cigarettes was not collected and thus we are unable to determine what effect this had on participants’ attitudes and behaviour. Furthermore, to ensure that the health warnings used on UK branded and Australian plain packs were matched, a warning demonstrating the effects of smoking on the foetus was chosen, as this was the only similar warning between the two countries. Although it could be argued that this is a female-specific warning, there was no clear evidence that the effects observed differed between males and females.

Second, smokers in the present study only used the branded or plain cigarette pack cigarettes for a single day, allowing us to only investigate the effects of plain packaging after first exposure. While previous experiments investigating the impact of cigarette packaging on attitudes to smoking assessed behaviour for up to two weeks, these studies suffered relatively high attrition [6]. The shorter trial period used here ensured minimal attrition, and therefore reduced the risk of bias due to selective drop-out. A longer trial period would have also precluded the use of the smoking topography monitor and the use of the costly imported Australian cigarettes – two major strengths of the present study. Nevertheless, a longer trial would be required to determine what effect plain cigarette packaging might have over a longer period, with repeated use. Indeed, any chances in actual smoking behaviour may only take effect over the longer term.

Third, as we only recruited adult daily smokers due to ethical considerations, our data say nothing about what effect plain cigarette packaging might have on non-smokers, smokers outside of our age inclusion criteria (18–40), or smokers who are attempting to quit smoking, despite research suggesting that plain packaging may be more effective among these groups [23,24]. We did not include these groups as we felt that it would be unethical to give smokers who wanted to quit a full pack of cigarettes to smoke and we used a relatively small age range of participants to increase the homogeneity of our sample. In addition, plain packaging is a population level intervention and not one targeted only at those planning on quitting. As we observed clear differences in attitudes to smoking in our sample of dependent adult smokers, it is possible that these effects might be even more pronounced among younger people and those smokers who are already considering quitting smoking.

Fourth, many participants were recruited from the staff and students at the University of Bristol, and one of the channels of recruitment was word-of-mouth, increasing the likelihood of contamination. However, measures such as asking all participants at the end of the experiment not to discuss the purpose with any other potential participants were taken, although compliance with this request was not directly assessed so some contamination between trial conditions is a possibility. In addition, as the majority of the participants were recruited from student populations, it is unknown to what extent the findings are generalisable to the wider population.

Fifth, the study was powered to detect a reduction in inhalation volume of 50 mL per cigarette, which is a relatively large effect in the context of smoking behaviour. Our study therefore lacked power to detect smaller but nevertheless important changes in smoking behaviour, such as number of cigarettes smoked, another of our primary outcome measures. However, the point estimates we observed suggest the possibility of a reduction in cigarettes smoked, but an increase in inhalation volume. Although we cannot draw conclusions from these data, given the wide confidence intervals associated with them, they are consistent with the known plasticity of smoking behaviour [25]. Finally, although the smoking topography monitors were used by participants in all conditions, using these monitors may have affected smoking behaviour. However, a three-item questionnaire regarding experiences of using the monitor did not observe any differences between participants randomised to the branded versus plain cigarette pack condition.

## Conclusions

This is the first randomised controlled trial of the effects of using genuine plain cigarette packs on actual smoking behaviour and related attitudes towards smoking. Our study provides evidence of the effect of first exposure to plain packaging on smoking behaviour and related attitudes to smoking and quitting. This first exposure acts as a precursor to later, longer-term effects on behaviour, such as quitting smoking. Although we found that plain cigarette packaging did not directly impact on smoking behaviour among regular smokers during the short time period of this trial, we observed that using a plain cigarette pack for a 24 hour period had clear effects on reducing experience of using the pack and ratings of the pack attributes and increased the impact of the health warning. A larger trial, with participants using the packs for a longer time period, would provide more detailed evidence on the impact of plain cigarette packaging on these attitudes to smoking, and whether they result in changes in smoking behaviour over the longer term. Nevertheless, these results add to the growing evidence base which suggests that plain cigarette packaging may be an effective tobacco control measure.

## Additional file

Additional file 1:
**Summary of linear regression results for final test day including results for individual questionnaire items.**

## Abbreviations

CO: Carbon monoxide
CI: Confidence interval
FTND: Fagerström Test for Nicotine Dependence
M: Mean
IEU: MRC Integrative Epidemiology Unit
QSU: Questionnaire of Smoking Urges
SE: Standard Error
SD: Standard Deviation
UK: United Kingdom
