# Supplementary material for: Effects of first exposure to plain cigarette packaging on smoking behaviour and attitudes: a randomised controlled study
Source: BMC Public Health. 2015 Mar 13;15:240. doi: 10.1186/s12889-015-1586-8 (PMC4394558; doi:10.1186/s12889-015-1586-8)
Supplement: Additional file 1: — Summary of linear regression results for final test day including results for individual questionnaire items. [file 12889_2015_1586_MOESM1_ESM.docx]

|  |  |  | Unadjusted | | | | Adjusted | | | |
| --- | --- | --- | --- | --- | --- | --- | --- | --- | --- | --- |
|  | Branded  Mean  (SE)  (*n* = 63) | Plain  Mean  (SE)  (*n* = 64) | B | Lower | Upper | P-value | B | Lower | Upper | P-value |
| Primary outcomes | | | | | | | | | | |
| Number of Cigarettes Smoked | 10.86  (0.45) | 10.34  (0.48) | -0.51 | -1.81 | +0.78 | 0.435 | -0.58 | -1.63 | +0.48 | 0.279 |
| Volume (mL) of Smoke Inhaled | 765.15  (62.41) | 817.26  (55.24) | +52.10 | -112.70 | +216.91 | 0.533 | +54.78 | -112.50 | +222.07 | 0.518 |
| Secondary Outcomes | | | | | | | | | | |
| **Experience of smoking** | **3.77**  **(0.09)** | **3.57**  **(0.10)** | **-0.20** | **-0.47** | **+0.07** | **0.140** | **-0.18** | **-0.45** | **+0.09** | **0.183** |
| Enjoyment: Not at all enjoyable (1)  – Very enjoyable (5) | 3.63  (0.11) | 3.25  (0.13) | -0.39 | -0.72 | -0.05 | 0.025 | -0.36 | -0.69 | -0.02 | 0.037 |
| Satisfaction: Not at all satisfying (1)  – Satisfying (5) | 3.76  (0.13) | 3.56  (0.13) | -0.20 | -0.56 | **+**0.16 | 0.271 | -0.18 | -0.54 | **+**0.18 | 0.312 |
| Acceptance: Unaccepted (1)  – Accepted (5) | 3.89  (0.11) | 3.89  (0.13) | 0.00 | -0.33 | **+**0.33 | 0.992 | 0.18 | -0.31 | **+**0.35 | 0.913 |
| **Experience of using the pack** | **4.63**  **(0.07)** | **4.13**  **(0.13)** | **-0.50** | **-0.80** | **-0.21** | **0.001** | **-0.52** | **-0.82** | **-0.22** | **0.001** |
| Embarrassment: Embarrassed (1)  – Not embarrassed (5) | 4.73  (0.08) | 4.19  (0.16) | -0.54 | -0.89 | -0.19 | 0.003 | -0.57 | -0.92 | -0.22 | 0.002 |
| Shame: Ashamed (1)  – Not ashamed (5) | 4.63  (0.09) | 4.03  (0.15) | -0.60 | -0.96 | -0.25 | 0.001 | -0.61 | -0.97 | -0.26 | 0.001 |
| Acceptance: Unaccepted (1)  – Accepted (5) | 4.52  (0.09) | 4.16  (0.13) | -0.37 | -0.68 | -0.05 | 0.022 | -0.21 | -0.69 | -0.06 | 0.022 |
| **Rating of pack attributes** | **3.52**  **(0.08)** | **1.91**  **(0.07)** | **-1.61** | **-1.82** | **-1.40** | **< 0.001** | **-1.59** | **-1.80** | **-1.39** | **< 0.001** |
| Style: Not stylish (1)  – Stylish (5) | 3.51  (0.13) | 1.38  (0.10) | -2.13 | -2.45 | -1.82 | <0.001 | -2.12 | -2.44 | -1.81 | <0.001 |
| Fashion: Unfashionable (1)  – Fashionable (5) | 3.11  (0.12) | 1.48  (0.09) | -1.63 | -1.94 | -1.32 | <0.001 | -1.61 | -1.92 | -1.30 | <0.001 |
| Cheapness: Cheap (1)  – Expensive (5) | 4.10  (0.14) | 2.55  (0.11) | -1.55 | -1.89 | -1.21 | <0.001 | -1.53 | -1.88 | -1.19 | <0.001 |
| Coolness: Uncool (1)  – Cool (5) | 3.03  (0.11) | 2.00  (0.11) | -1.03 | -1.34 | -0.73 | <0.001 | -1.00 | -1.30 | -0.70 | <0.001 |
| Attractiveness: Unattractive (1)  – Attractive (5) | 3.06  (0.13) | 1.48  (0.11) | -1.58 | -1.92 | -1.24 | <0.001 | -1.55 | -1.89 | -1.22 | <0.001 |
| Quality: Poor quality (1)  – Good quality (5) | 4.02  (0.10) | 2.97  (0.13) | -1.05 | -1.37 | -0.73 | <0.001 | -1.05 | -1.37 | -0.72 | <0.001 |
| Appeal: Unappealing (1)  – Appealing (5) | 3.83  (0.09) | 1.50  (0.09) | -2.33 | -2.57 | -2.08 | <0.001 | -2.32 | -2.56 | -2.08 | <0.001 |
| **Rating of the health warning** | **3.92**  **(0.11)** | **4.41**  **(0.08)** | **+0.49** | **+0.23** | **+0.76** | **< 0.001** | **+0.51** | **+0.24** | **+0.78** | **< 0.001** |
| Noticing: Hardly noticeable (1)  – Very noticeable (5) | 3.35  (0.18) | 4.61  (0.09) | **+**1.26 | **+**0.87 | **+**1.65 | <0.001 | **+**1.28 | **+**0.89 | **+**1.67 | <0.001 |
| Seriousness: Not serious (1)  – Very serious (5) | 3.92  (0.14) | 4.41  (0.10) | **+**0.49 | **+**0.15 | **+**0.82 | 0.005 | **+**0.51 | **+**0.18 | **+**0.84 | 0.003 |
| Believability: Not believable (1)  – Believable (5) | 4.21  (0.10) | 4.25  (0.11) | **+**0.04 | -0.25 | **+**0.34 | 0.768 | **+**0.06 | -0.24 | **+**0.35 | 0.698 |
| Awareness of health risks: Not at all aware (1)  – Very aware (5) | 4.21  (0.14) | 4.39  (0.09) | **+**0.18 | -0.14 | **+**0.51 | 0.261 | **+**0.20 | -0.13 | **+**0.53 | 0.228 |
| **Change in behaviour**  Did you: | **2.06**  **(0.21)** | **2.20**  **(0.20)** | **+0.14** | **-0.43** | **+0.71** | **0.629** | **+0.11** | **-0.45** | **+0.68** | **0.695** |
| **Stub out a cigarette early** | 0.37  (0.06) | 0.41  (0.06) | **+**0.04 | -0.13 | **+**0.21 | 0.637 | **+**0.03 | -0.14 | **+**0.20 | 0.723 |
| **Forgo a cigarette** | 0.30  (0.06) | 0.33  (0.06) | **+**0.03 | -0.14 | **+**0.19 | 0.750 | **+**0.03 | -0.14 | **+**0.19 | 0.744 |
| **Keep the pack out of sight** | 0.13  (0.04) | 0.28  (0.06) | **+**0.15 | **+**0.01 | **+**0.29 | 0.031 | **+**0.15 | **+**0.01 | **+**0.29 | 0.031 |
| **Cover the pack** | 0.02  (0.02) | 0.09  (0.04) | **+**0.08 | 0.00 | **+**0.16 | 0.055 | **+**0.08 | 0.00 | **+**0.16 | 0.044 |
| **Smoke less around others** | 0.21  (0.05) | 0.28  (0.06) | **+**0.08 | -0.08 | **+**0.23 | 0.330 | **+**0.07 | -0.09 | **+**0.22 | 0.401 |
| **Think about cutting down** | 0.46  (0.06) | 0.31  (0.06) | -0.15 | -0.32 | **+**0.02 | 0.088 | -0.15 | -0.32 | **+**0.02 | 0.079 |
| **Think about quitting in the next few weeks** | 0.11  (0.04) | 0.06  (0.03) | -0.05 | -0.15 | **+**0.05 | 0.334 | -0.05 | -0.15 | **+**0.05 | 0.332 |
| **Thinking about quitting within a year** | 0.48  (0.06) | 0.44  (0.06) | -0.04 | -0.22 | **+**0.14 | 0.665 | -0.04 | -0.22 | **+**0.13 | 0.627 |
| **Attitudes to plain packs**  Do you think plain packaging would: | **6.66**  **(0.30)** | **6.21**  **(0.05)** | **-0.45** | **-1.27** | **+0.38** | **0.285** | **-0.39** | **-1.22** | **+0.44** | **0.350** |
| Make you smoke fewer cigarettes?:  Not at all (1)  – Very much so (4) | 2.05  (0.12 | 1.86  (0.12) | -0.19 | -0.52 | **+**0.15 | 0.266 | -0.17 | -0.49 | **+**0.16 | 0.313 |
| Help you quit smoking?: Not at all (1)  – Very much so (4) | 1.95  (0.12) | 1.73  (0.11) | -0.22 | -0.55 | **+**0.11 | 0.195 | -0.21 | -0.54 | **+**0.13 | 0.222 |
| Prevent children from starting smoking?: Not at all (1)  – Very much so (4) | 2.87  (0.12) | 2.66  (0.12) | -0.22 | -0.54 | **+**0.11 | 0.190 | -0.19 | -0.52 | 0.13 | 0.243 |
| **Experience of using monitor** | **3.80**  **(0.12)** | **3.98**  **(0.11)** | **0.18** | **-0.13** | **+0.49** | **0.246** | **0.19** | **-0.11** | **0.49** | **0.217** |
| Embarrassment: Embarrassed (1)  – Not embarrassed (5) | 3.17  (0.17) | 3.55  (0.16) | 0.37 | -0.09 | **+**0.84 | 0.116 | 0.37 | -0.08 | 0.82 | 0.105 |
| Shame: Ashamed (1)  – Not ashamed (5) | 4.37  (0.12) | 4.47  (0.09) | 0.10 | -0.20 | **+**0.41 | 0.498 | 0.11 | -0.19 | 0.41 | 0.465 |
| Acceptance: Unaccepted (1)  – Accepted (5) | 3.86  (0.13) | 3.01  (0.12) | 0.05 | -0.30 | **+**0.40 | 0.783 | 0.06 | -0.29 | 0.41 | 0.731 |
